# Supplementary figures and images for: Salt marsh sediment bacterial communities maintain original population structure after transplantation across a latitudinal gradient
Source: PeerJ. 2018 May 1;6:e4735. doi: 10.7717/peerj.4735 (PMC5935077; doi:10.7717/peerj.4735)

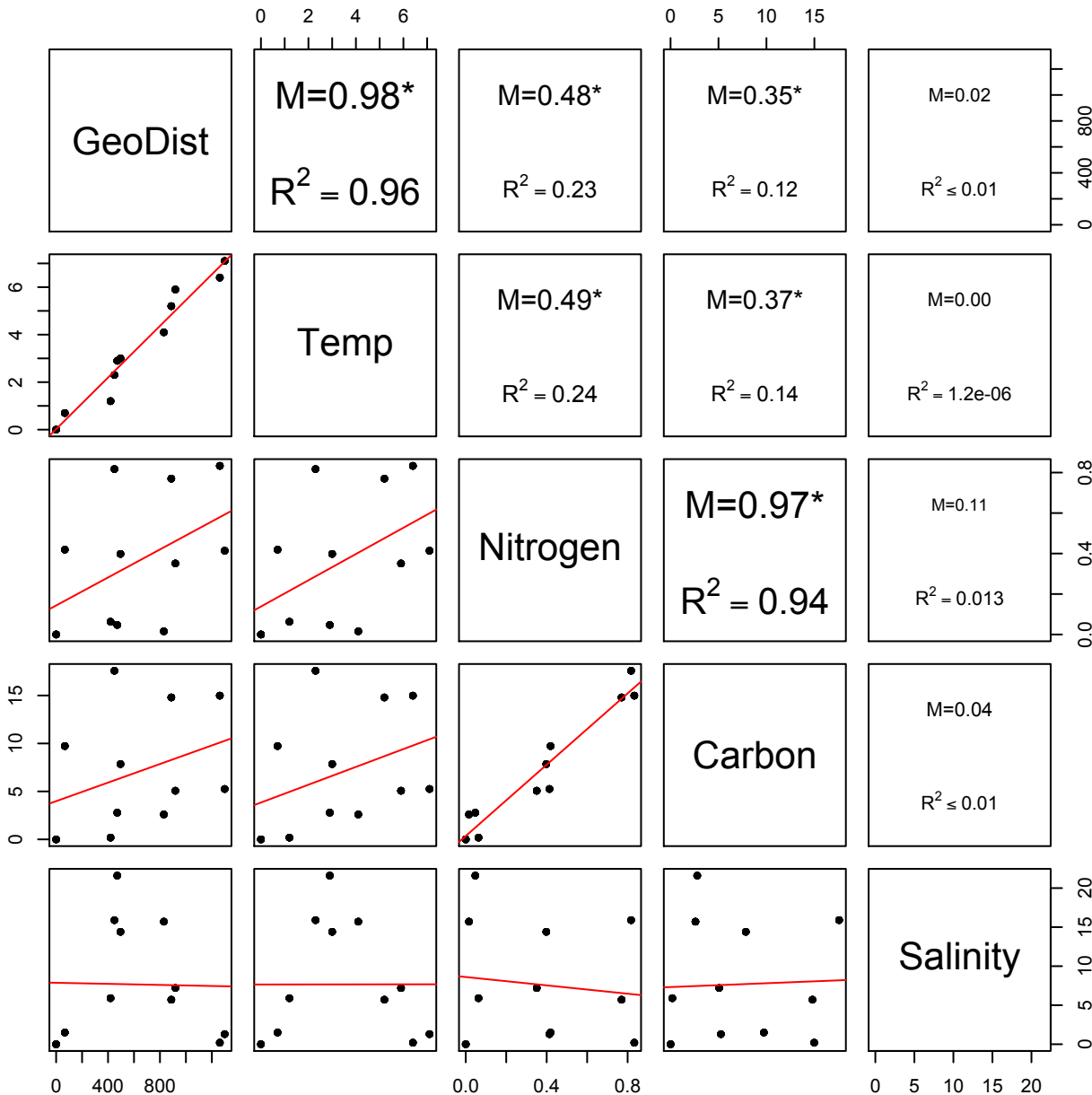

Supplement: Supplemental Information 1 — Each box above or below the diagonal is a pairwise analysis. The upper triangle shows the results for each pair with font size scaled to correlation strength. The lower triangle contains plots of the best fit linear regression. [file peerj-06-4735-s001.pdf]

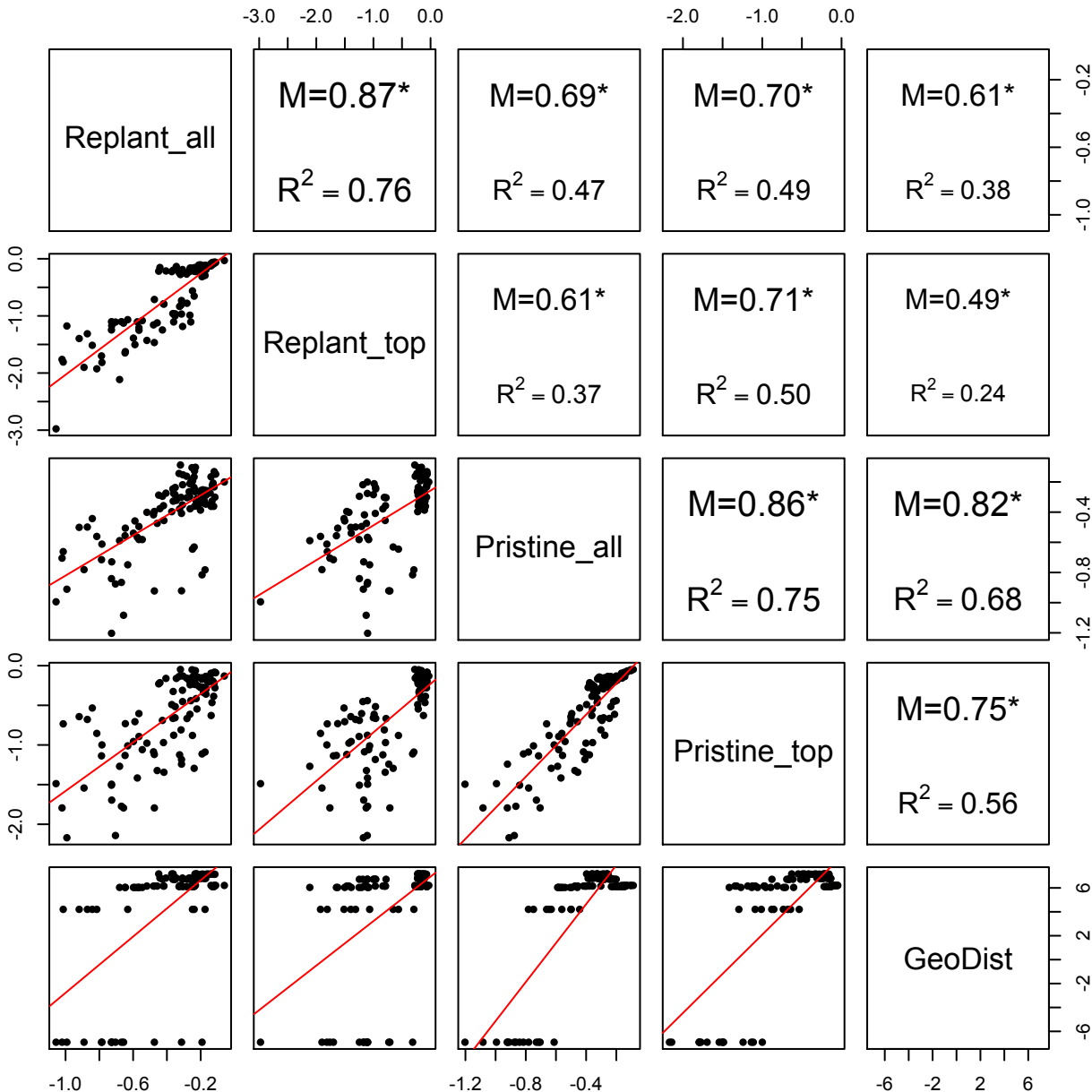

Supplement: Supplemental Information 2 — Each box above or below the diagonal is a pairwise analysis. The upper triangle shows the results for each pair with font size scaled to correlation strength. The lower triangle contains plots of the best fit linear regression. [file peerj-06-4735-s002.pdf]

**A****Stress = 0.29**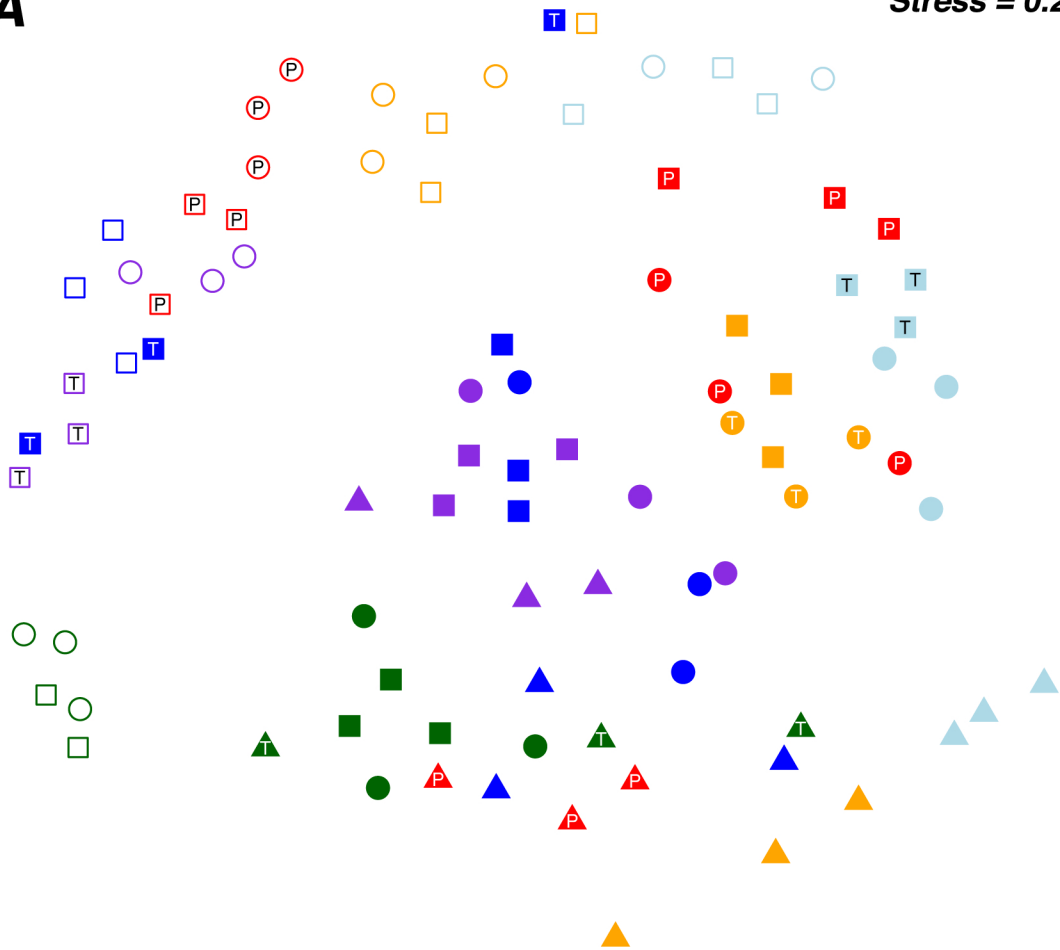**B****Stress = 0.17**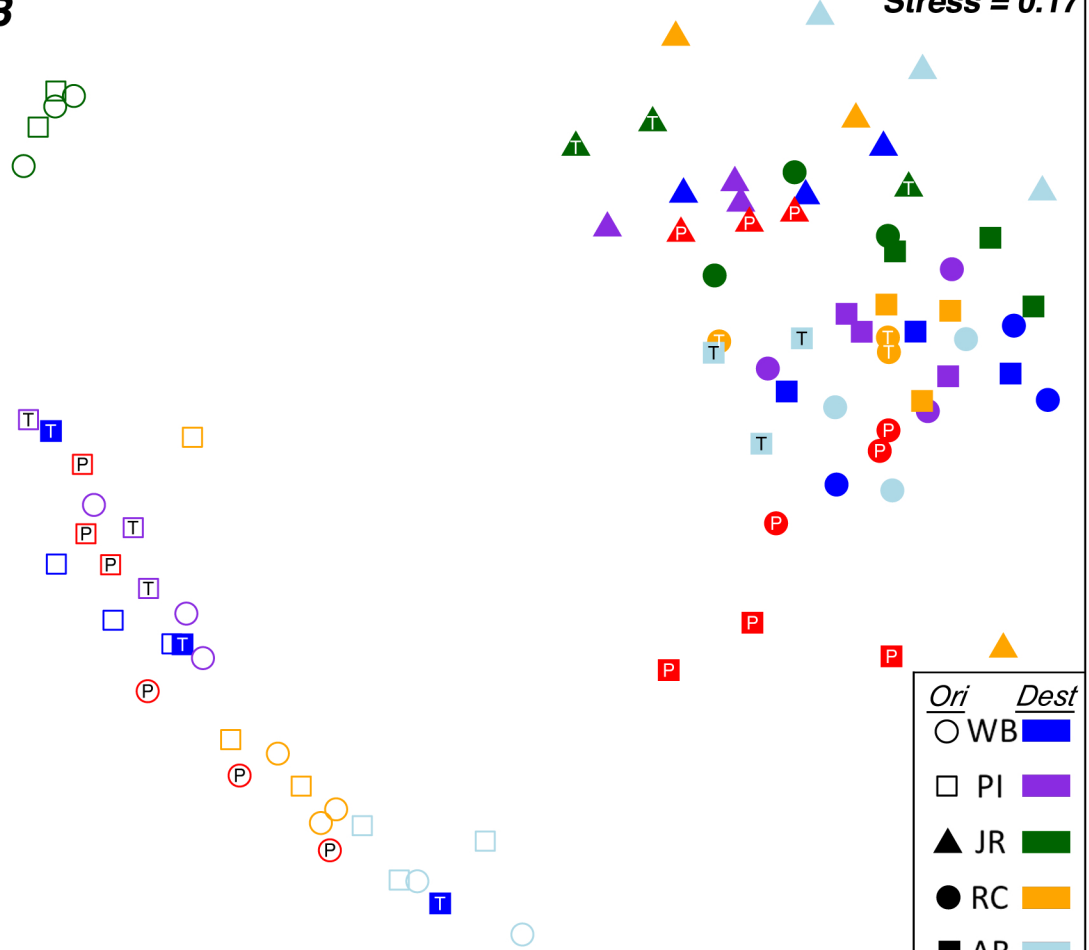

Supplement: Supplemental Information 3 — (A) All 16S rRNA gene OTUs for all samples; and (B) Top ten most abundant 16S rRNA gene OTUs for all samples. Symbols with a “P” are pristine control samples and symbols with a “T” are transplant control samples. Pristine control symbols are additionally colored ‘red’. All others are experimental transplant samples. Symbol shapes indicate origin sites while colors indicate destination sites for all samples. [file peerj-06-4735-s003.pdf]

***Stress = 0.31***

- WB
- PI
- ▲ JR
- RC
- AB

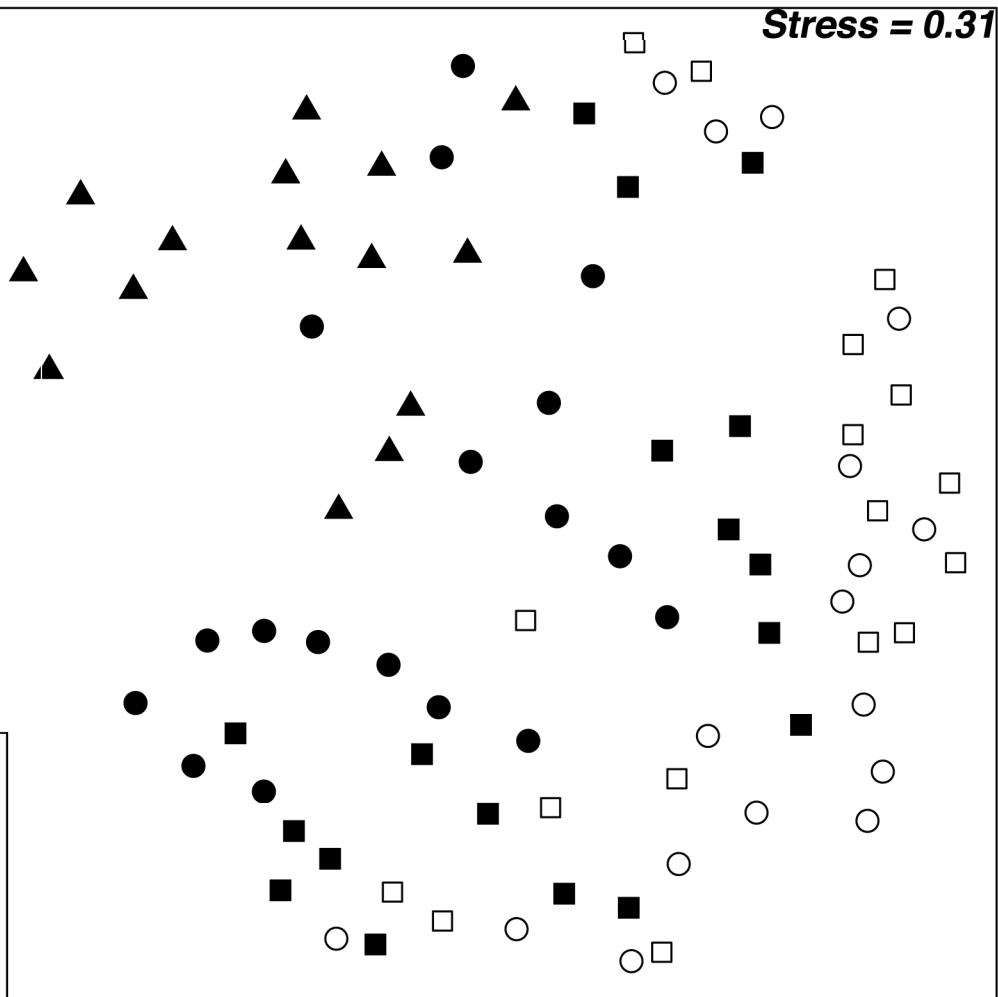

Supplement: Supplemental Information 4 — Rare taxa defined by those 16S rRNA gene OTUs occurring <5% across all samples. Symbols refer to each samples origin site. [file peerj-06-4735-s004.pdf]
