# Supplementary material for: Salt marsh sediment bacterial communities maintain original population structure after transplantation across a latitudinal gradient
Source: PeerJ. 2018 May 1;6:e4735. doi: 10.7717/peerj.4735 (PMC5935077; doi:10.7717/peerj.4735)
Supplement: Supplemental Information 5 — Indicator 16S rRNA gene OTUs determined by pairwise linear regressions between relative abundance of each OTU and degree of environmental change. Only four OTUs above the red line met the p-value and slope thresholds (p-value < 5e-9, R-squared > 0.40). OTU percent abundance is the fraction of all sequences that belong to that OTU. List is truncated and does not show low significance results below R-squared of [0.14]. [file peerj-06-4735-s005.pdf]

QIIME OTU#	Indicator OTU Taxonomy	Env Variable	Slope	R-squared	p-value
21858	Bacteria; Chloroflexi; Anaerolineae; Anaerolineales; Anaerolinaceae	deltaN	-1.64	0.59	2.34E-15
21858	Bacteria; Chloroflexi; Anaerolineae; Anaerolineales; Anaerolinaceae	deltaTemp	4.01	0.46	4.94E-11
32074	Bacteria; Proteobacteria; Gammaproteobacteria; Vibrionales; Vibrionaceae; Vibrio	deltaTemp	-3.53	0.41	8.66E-10
13670	Bacteria; Proteobacteria; Deltaproteobacteria; Desulfobacterales; Desulfobacteraceae	deltaN	-1.22	0.41	1.29E-09
39777	Bacteria; Chloroflexi; Anaerolineae; Anaerolineales; Anaerolinaceae; Bellilinea	deltaTemp	3.55	0.37	8.55E-09
32074	Bacteria; Proteobacteria; Gammaproteobacteria; Vibrionales; Vibrionaceae; Vibrio	deltaN	1.21	0.37	8.91E-09
18564	Bacteria; Acidobacteria; Acidobacteria; Acidobacteriales; Acidobacteriaceae	deltaN	-1.12	0.36	1.78E-08
18564	Bacteria; Acidobacteria; Acidobacteria; Acidobacteriales; Acidobacteriaceae	deltaTemp	2.86	0.30	4.23E-07
39777	Bacteria; Chloroflexi; Anaerolineae; Anaerolineales; Anaerolinaceae; Bellilinea	deltaN	-1.14	0.30	5.27E-07
31042	Bacteria; Proteobacteria; Gammaproteobacteria; Thiotrichales; Piscirickettsiaceae; Mariprofundus	deltaSalinity	-2.71	0.29	6.97E-07
50561	Bacteria; Chloroflexi; Anaerolineae; Anaerolineales	deltaTemp	4.46	0.29	7.88E-07
6580	Bacteria; Bacteroidetes; Sphingobacteria; Sphingobacteriales	deltaN	-1.52	0.28	1.59E-06
15121	Bacteria; WS3	deltaN	-1.14	0.27	2.59E-06
7363	Bacteria; Planctomycetes; Phycisphaerae	deltaSalinity	-0.94	0.27	2.73E-06
62033	Bacteria; Chloroflexi; Anaerolineae; Anaerolineales	deltaN	-1.36	0.26	3.90E-06
62033	Bacteria; Chloroflexi; Anaerolineae; Anaerolineales	deltaTemp	3.75	0.26	4.60E-06
13670	Bacteria; Proteobacteria; Deltaproteobacteria; Desulfobacterales; Desulfobacteraceae	deltaTemp	2.69	0.26	4.65E-06
47211	Bacteria; Proteobacteria; Deltaproteobacteria; Desulfobacterales; Desulfobacteraceae	deltaSalinity	-0.86	0.25	6.21E-06
28760	Bacteria; Proteobacteria; Deltaproteobacteria; Desulfarculales; Desulfarculaceae	deltaTemp	4.55	0.25	7.46E-06
1399	Bacteria; Bacteroidetes; Sphingobacteria; Sphingobacteriales	deltaN	-1.57	0.24	8.90E-06
62643	Bacteria	deltaN	-0.86	0.22	2.70E-05
50561	Bacteria; Chloroflexi; Anaerolineae; Anaerolineales	deltaN	-1.39	0.22	2.89E-05
7363	Bacteria; Planctomycetes; Phycisphaerae	deltaN	-0.87	0.22	2.96E-05
46703	Bacteria; Proteobacteria; Deltaproteobacteria; Desulfuromonadales; Desulfuromonadaceae; Desulfuromusa	deltaTemp	3.82	0.22	2.98E-05
55979	Bacteria; Proteobacteria; Deltaproteobacteria; Desulfobacterales; Desulfobulbaceae	deltaTemp	4.01	0.22	3.07E-05
32072	Bacteria; Proteobacteria; Gammaproteobacteria; Alteromonadales; Shewanellaceae; Shewanella	deltaN	1.41	0.22	3.28E-05
23473	Bacteria; Proteobacteria; Deltaproteobacteria; Desulfobacterales; Desulfobacteraceae	deltaN	-0.97	0.21	4.47E-05
20646	Bacteria; Chlorobi; Chlorobia; Chlorobiales	deltaTemp	4.56	0.21	4.98E-05
20646	Bacteria; Chlorobi; Chlorobia; Chlorobiales	deltaN	-1.64	0.21	5.42E-05
35044	Bacteria; Deferribacteres; Deferribacteres; Deferribacterales; Unassigned; Caldithrix	deltaSalinity	-1.26	0.21	5.46E-05
34997	Bacteria; Proteobacteria; Deltaproteobacteria; Syntrophobacterales; Syntrophaceae; Desulfobacca	deltaTemp	3.30	0.21	5.64E-05
52530	Bacteria; WS3	GeoDist	-2.36	0.20	5.89E-05
10059	Bacteria; Proteobacteria; Deltaproteobacteria; Desulfarculales; Desulfarculaceae	deltaN	-0.69	0.20	6.09E-05
23410	Bacteria; Proteobacteria; Gammaproteobacteria; Acidithiobacillales; Acidithiobacillaceae; Acidithiobacillus	deltaSalinity	-1.05	0.20	6.50E-05
50299	Bacteria; Proteobacteria; Deltaproteobacteria; Desulfobacterales; Desulfobacteraceae	deltaN	-1.00	0.20	6.84E-05
9333	Bacteria; Deferribacteres; Deferribacteres; Deferribacterales; Unassigned; Caldithrix	deltaN	-0.87	0.20	7.14E-05
15121	Bacteria; WS3	deltaTemp	2.69	0.19	9.60E-05
28853	Bacteria; Chloroflexi; Dehalococcoidetes; Unassigned; Unassigned; Dehalogenimonas	deltaTemp	2.24	0.19	9.81E-05
10080	Bacteria; Bacteroidetes; Sphingobacteria; Sphingobacteriales	deltaSalinity	-1.18	0.19	1.07E-04
28760	Bacteria; Proteobacteria; Deltaproteobacteria; Desulfarculales; Desulfarculaceae	deltaN	-1.43	0.19	1.28E-04
45571	Bacteria; Proteobacteria; Gammaproteobacteria; Acidithiobacillales; Acidithiobacillaceae; Acidithiobacillus	deltaSalinity	-1.61	0.19	1.30E-04
32068	Bacteria; Proteobacteria; Gammaproteobacteria; Oceanospirillales; Oceanospirillaceae; Marinomonas	deltaN	1.31	0.19	1.37E-04
42445	Bacteria; Planctomycetes; Phycisphaerae	deltaTemp	4.76	0.18	1.55E-04
27155	Bacteria; Deferribacteres; Deferribacteres; Deferribacterales; Unassigned; Caldithrix	deltaSalinity	-1.88	0.18	1.66E-04
13729	Bacteria; Planctomycetes; Phycisphaerae	deltaTemp	2.15	0.18	1.91E-04
53715	Bacteria; Bacteroidetes; Sphingobacteria; Sphingobacteriales	deltaSalinity	-0.99	0.18	2.10E-04
28853	Bacteria; Chloroflexi; Dehalococcoidetes; Unassigned; Unassigned; Dehalogenimonas	deltaN	-0.77	0.18	2.12E-04
27405	Bacteria; Acidobacteria; Acidobacteria; Acidobacteriales; Acidobacteriaceae	deltaN	-0.63	0.17	2.70E-04
1399	Bacteria; Bacteroidetes; Sphingobacteria; Sphingobacteriales	deltaTemp	3.63	0.17	2.83E-04
59708	Bacteria; Proteobacteria; Deltaproteobacteria; Desulfobacterales; Desulfobacteraceae	deltaSalinity	-1.22	0.17	2.83E-04
42445	Bacteria; Planctomycetes; Phycisphaerae	deltaN	-1.64	0.17	3.15E-04
6585	Bacteria; Spirochaetes; Spirochaetes; Spirochaetales; Spirochaetaceae; Spirochaeta	deltaN	-0.52	0.17	3.20E-04
45571	Bacteria; Proteobacteria; Gammaproteobacteria; Acidithiobacillales; Acidithiobacillaceae; Acidithiobacillus	deltaN	-1.56	0.17	3.39E-04
23473	Bacteria; Proteobacteria; Deltaproteobacteria; Desulfobacterales; Desulfobacteraceae	deltaTemp	2.38	0.17	3.52E-04
53114	Bacteria; Chloroflexi; Dehalococcoidetes; Unassigned; Unassigned; Dehalogenimonas	deltaTemp	2.18	0.16	3.75E-04
22720	Bacteria; Firmicutes; Clostridia; Clostridiales; Ruminococcaceae; Acetivibrio	deltaSalinity	-0.57	0.16	3.78E-04
10059	Bacteria; Proteobacteria; Deltaproteobacteria; Desulfarculales; Desulfarculaceae	deltaTemp	1.70	0.16	4.06E-04
50299	Bacteria; Proteobacteria; Deltaproteobacteria; Desulfobacterales; Desulfobacteraceae	deltaSalinity	-0.87	0.16	4.13E-04
10348	Bacteria; Proteobacteria; Gammaproteobacteria; Chromatiales; Chromatiaceae; Nitrosococcus	deltaN	-1.42	0.16	4.63E-04
6580	Bacteria; Bacteroidetes; Sphingobacteria; Sphingobacteriales	deltaTemp	3.18	0.16	4.95E-04
8983	Bacteria; Chlorobi; Chlorobia; Chlorobiales	deltaSalinity	-1.39	0.15	6.84E-04
13729	Bacteria; Planctomycetes; Phycisphaerae	deltaN	-0.71	0.15	7.70E-04
43234	Bacteria; Proteobacteria; Deltaproteobacteria; Desulfarculales; Desulfarculaceae	deltaTemp	1.41	0.15	7.87E-04
50708	Bacteria; Chloroflexi; Dehalococcoidetes; Unassigned; Unassigned; Dehalogenimonas	deltaTemp	1.57	0.15	7.94E-04
53715	Bacteria; Bacteroidetes; Sphingobacteria; Sphingobacteriales	deltaN	-0.92	0.15	8.38E-04
9333	Bacteria; Deferribacteres; Deferribacteres; Deferribacterales; Unassigned; Caldithrix	deltaTemp	2.05	0.15	8.53E-04
40391	Bacteria; Spirochaetes; Spirochaetes; Spirochaetales; Spirochaetaceae; Spirochaeta	deltaN	-0.91	0.15	8.82E-04
46703	Bacteria; Proteobacteria; Deltaproteobacteria; Desulfuromonadales; Desulfuromonadaceae; Desulfuromusa	deltaN	-1.12	0.14	8.92E-04
29982	Bacteria; Proteobacteria; Deltaproteobacteria; Desulfarculales; Desulfarculaceae	deltaTemp	2.49	0.14	9.15E-04
31973	Bacteria; Proteobacteria; Deltaproteobacteria; Desulfarculales; Desulfarculaceae	deltaN	-0.39	0.14	9.17E-04
28873	Bacteria; Spirochaetes; Spirochaetes; Spirochaetales; Spirochaetaceae; Spirochaeta	deltaN	-0.64	0.14	9.90E-04
59056	Bacteria; Proteobacteria; Deltaproteobacteria; Desulfobacterales; Desulfobacteraceae	deltaSalinity	-1.09	0.14	9.90E-04

•••
